# Development of Enthesopathies and Joint Structural Damage in a Murine Model of X-Linked Hypophosphatemia

**DOI:** 10.3389/fcell.2020.00854

**Published:** 2020-09-22

**Authors:** Carole-Anne Faraji-Bellée, Axelle Cauliez, Benjamin Salmon, Olivier Fogel, Volha Zhukouskaya, Aurélie Benoit, Thorsten Schinke, Christian Roux, Agnès Linglart, Corinne Miceli-Richard, Catherine Chaussain, Karine Briot, Claire Bardet

**Affiliations:** ^1^Université de Paris, Laboratory Orofacial Pathologies, Imaging and Biotherapies UR 2496, Dental School, Montrouge, France; ^2^APHP, Reference Center for Rare Disorders of the Calcium and Phosphate Metabolism, Dental Medicine Department, Bretonneau Hospital, Paris, France; ^3^Department of Rheumatology, Cochin Hospital, Université de Paris, Reference Center for Rare Disorders of the Calcium and Phosphate Metabolism, and Reference Center for Rare Genetic Bone Diseases, Cochin Hospital, Paris, France; ^4^APHP, Reference Center for Rare Disorders of the Calcium and Phosphate Metabolism, Filière OSCAR and Platform of Expertise for Rare Diseases Paris-Sud, Bicêtre Paris-Sud Hospital, Le Kremlin Bicêtre, France; ^5^Université de Paris, URB2I, UR 4462, Dental School, Montrouge, France; ^6^Department of Osteology and Biomechanics, University Medical Center Hamburg Eppendorf, Hamburg, Germany; ^7^APHP, Department of Endocrinology and Diabetology for Children, Bicêtre Paris Sud Hospital, Le Kremlin-Bicêtre, France; ^8^Paris Sud – Paris Saclay University, Faculté de Médecine, Le Kremlin – Bicêtre, France

**Keywords:** *Hyp* mouse, natural history, enthesophyte, joint alteration, XLH

## Abstract

X-linked hypophosphatemia (XLH) is characterized by rickets and osteomalacia, caused by inactivating mutations in the Phosphate-regulating endopeptidase homolog X-linked (PHEX) gene. With aging, adult patients develop paradoxical heterotopic calcifications of tendons and ligaments at their insertion sites (enthesophytes), and joint alterations. Understanding the progression of this structural damage that severely affects patients’ quality of life will help to improve the management of XLH. Here, we characterized the occurrence of enthesophytes and joint alterations through a 12 month *in vivo* micro-CT follow-up in the *Hyp* mouse, a murine model of XLH (*n* = 5 mice per group). Similar to adult patients with XLH, *Hyp* mice developed calcaneal enthesophytes, hip joint alterations, erosions of the sacroiliac joints and periarticular calcifications. These lesions were already present at month 3 and gradually worsened over time. In sharp contrast, no abnormalities were observed in control mice at early time points. Histological analyses confirmed the presence of bone erosions, calcifications and expansion of mineralizing enthesis fibrocartilage in *Hyp* mice and their absence in controls and suggested that new bone formation is driven by altered mechanical strain. Interestingly, despite a strong deformation of the curvature, none of the *Hyp* mice displayed enthesophyte at the spine. Peripheral enthesophytes and joint alterations develop at the early stages of the disease and gradually worsen overtime. Overall, our findings highlight the relevance of this preclinical model to test new therapies aiming to prevent bone and joint complications in XLH.

## Introduction

**FIGURE 1:**
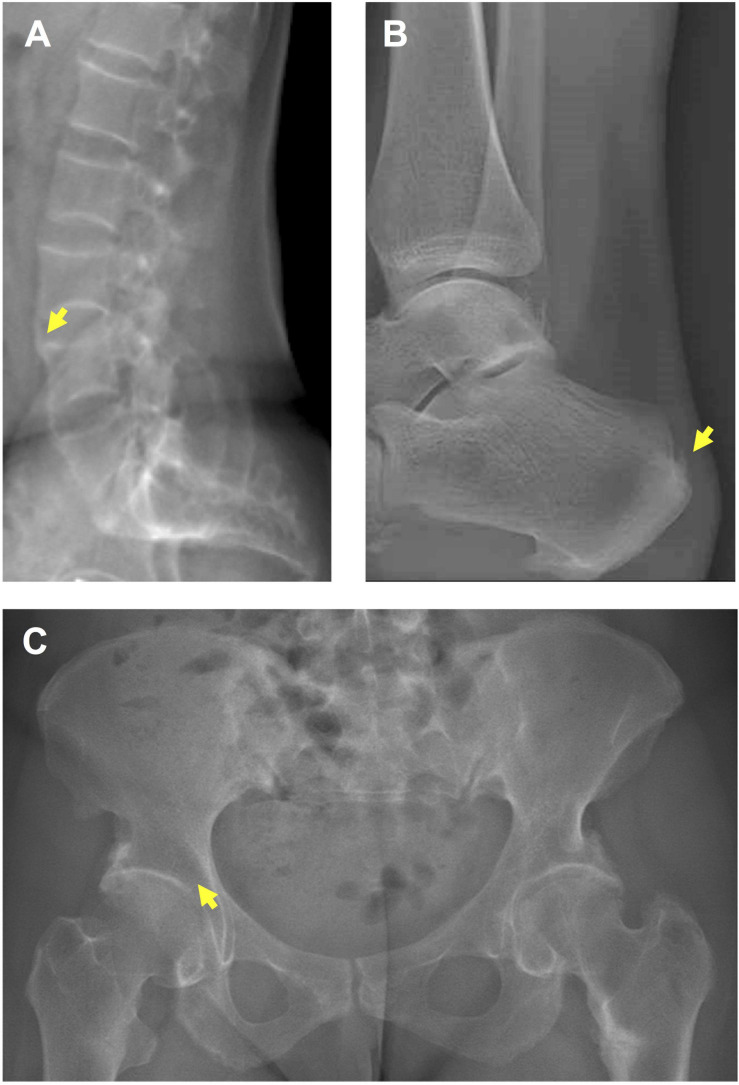
Enthesopathies and osteoarthritis in a 36 year-old female patient with XLH. **(A)** Spinal enthesophytes (yellow arrow). **(B)** Enthesophyte (yellow arrow) on the Achille’s tendon. **(C)** Early osteoarthritis (yellow arrow).

Mineralization defects and paradoxical mineralization of entheses are the hallmarks of X-linked hypophosphatemia (XLH) (Polisson et al., 1985; Reid et al., 1989), a rare skeletal disease caused by inactivating mutations in the phosphate-regulating endopeptidase homolog X-linked (*PHEX*) gene (Sabbagh et al., 2000; Gaucher et al., 2009). Mineralization defects manifest in XLH children as rickets with severe skeletal deformities and short stature (Wharton and Bishop, 2003; Carpenter et al., 2011; Glass et al., 2011; Fuente et al., 2017; Vakharia et al., 2018). In adulthood, the burden of disease is related to musculoskeletal symptoms due to osteomalacia (de Menezes Filho et al., 2006), osteoarthritis, and lower-limb muscle weakness, but also ossification of entheses (enthesophytes), this latter feature being a strong determinant of impairment in quality of life (QoL) in this population (Schott and Wills, 1976; Reid et al., 1989; Sullivan et al., 1992; Amora et al., 2006; Bergwitz and Juppner, 2009; Che et al., 2016; Skrinar et al., 2019). Enthesophytes form on the spine, peripheral joints and Achilles tendons, and are responsible for chronic pain, joint limitations, stiffness and disability (Figure 1; Che et al., 2016).

The current medical treatment, which is commonly prescribed from early childhood to the end of growth, seeks to address the deficiencies associated with this disease, i.e., oral phosphorus supplementation with multiple daily intakes to compensate for renal phosphate wasting and active vitamin D analogs (alfacalcidol or calcitriol) to counteract the 1,25-(OH)_2_-vitamin D deficiency (Glorieux et al., 1980; Friedman et al., 1993; Makitie et al., 2003; Liu et al., 2016). Nonetheless, despite this treatment during growth, musculoskeletal symptoms due to enthesopathies and osteoarthritis remain the main manifestations in the clinical progression of XLH (Carpenter, 2012; Linglart et al., 2014; Connor et al., 2015). It is unclear whether or not enthesophyte formation can be modified by using phosphate and vitamin D analogs. In an observational cross-sectional study in 52 adults with XLH, neither the presence nor the absence of enthesopathies was associated with conventional treatment of XLH, whatever the treatment duration (Connor et al., 2015). The pathogenesis underlying the development and progression of enthesopathies and osteoarthritis in patients with XLH has yet to be investigated.

The hypophosphatemic (*Hyp*) mouse, a murine model of XLH that harbors a spontaneous *Phex* mutation, recapitulates biochemical and skeletal features of XLH (Eicher et al., 1976; Strom et al., 1997). Although the expansion of mineralizing enthesis fibrocartilage of the calcaneus and osteoarthropathy of the tibial articular cartilage have been described (Liang et al., 2009; Liu et al., 2018), there are no data on spinal and other peripheral features in this model. Thus, in this study, we sought to describe the natural history of enthesopathies and joint alterations in XLH, using a 12 month (M) longitudinal follow-up of *Hyp* mice.

## Materials and Methods

### Mouse Model

The *Hyp* mouse model B6.Cg-Phex Hyp/J was obtained from the Jackson Laboratory (# 000528). Heterozygous breeding was carried out and tail snips were collected for genotyping. DNA was extracted from the snips using a DNeasy Blood and Tissue Kit (Qiagen, France) and the genotype was determined by PCR using primers for *Phex* (Sheen et al., 2012). Male wild-type (WT) and *Hyp* littermates were used for this study. Chemical methods were used for mice sacrifice according to the ethical protocol approved by the Animal Care Committee of French Veterinary Services (DPP Haut de Seine, France: agreement number C-9204901).

All mice were housed under conditions of controlled temperature (23 ± 2°C) and light (12:12 h light/dark cycle), with unlimited access to water and standard pelleted food (1.20% calcium and 0.83% phosphorus, rodent diet 3800PMS10, Provimi Kliba, Kaiseraugst, Switzerland).

### X-ray Micro-Computed Tomography (Micro-CT) Acquisition and Analysis

Male WT and *Hyp* mice (*n* = 5 per group) were scanned at M3, 6, 9 and 12 using a high-resolution X-ray micro-computer tomography (micro-CT) system (Quantum FX Caliper, Life Sciences, Perkin Elmer, Waltham, MA) at the Life Imaging Platform (PIV), UR 2496, Montrouge, France. Standard acquisition settings were used (90 kV, 160 mA) and scans were performed with a field of view alternately focused on the right hind paw (180 s scan time, 20 μm^3^ voxel size) or hip (120 s, 50 μm^3^), or covering the full body (36 s, 236 μm^3^). Micro-CT data sets were analyzed using the built-in multiplanar reconstruction tool, OsiriX 5.8 (Pixmeo, Switzerland) in order to generate a series of images aligned over time for each anatomical region of each animal. Axial and coronal images of the sacroiliac joints and hip joints, sagittal images of the spine, and axial and sagittal images of the hind paw were reconstructed. The analysis was focused on these areas because they are the most common sites of structural involvement in adult patients with XLH.

Two rheumatologists specialized in the field of rare bone diseases and bone inflammatory diseases assessed hip joint alterations (defined as the presence of osteophytes on joint margins or narrowing of the joint space or altered shape of the bone ends), and enthesopathies (defined as new bone formation from the enthesis) on spine or paw, erosion of the sacroiliac joint and periarticular calcification. Both readers were blind to the status of the mouse (*Hyp* vs. WT) but were aware of the different analysis time points (M3, M6, M9, and M12). A semi-quantitative score was established, ranging from 0 (normal) to 3 (most severe feature assessed) for spinal enthesophytes and joint evaluation and from 0 (absent) to 2 (present) for peripheral calcifications and enthesophytes (see Table 1). When applicable, both sides were scored and a resulting mean was calculated. In the follow-up score, up to 1 point could be added in cases of a 50% increase in the size of the enthesophyte or calcification between two consecutive time points. A composite score (from 0 to 18) was built based on these parameters for each time point (see reading grid in Table 1). Observers were calibrated during a preliminary 3 h training session on micro-CT reading. In the event of disagreement between the two readers, a third also analyzed the image and a consensus was reached between the three readers.

**TABLE 1:** Scoring grid of enthesopathies, erosions, ossifications, and narrowing of joint.

**Initial scoring**			**Follow-up scoring**		
**Variables**	**Score**	**Description**	**Variables**	**Score**	**Description**
Erosion of the sacroiliac joints	0	Normal	Erosion of the sacroiliac joints		Same as initial scoring
	1	Doubtful			
	2	<25% of the articular surface area affected			
	2.25	≥25% to <50% of the articular surface area affected			
	2.5	≥50% to <75% of the articular surface area affected			
	2.75	≥75% to <100% of the articular surface area affected			
	3	All the articular surface area affected			
Narrowing of the hip joint space	0	Normal	Narrowing of the hip joint space		Same as initial scoring
	1	Doubtful			
	2	< 25% pinching			
	2.25	≥25% to <50% pinching			
	2.5	≥50% to <75% pinching			
	2.75	≥75% to <100% pinching			
	3	Complete pinching			
Spinal enthesophytes	0	Normal	Spinal enthesophytes		Same as initial scoring
	1	Doubtful			
	2	<25% of the line spacing			
	2.25	≥25% to <50% of the line spacing			
	2.5	≥50% to <75% of the line spacing			
	2.75	≥75% to <100% of the line spacing			
	3	Full bridge			
Calcaneal enthesophytes	0	Absent	Calcaneal enthesophyte size	0	stable
	1	Doubtful		Add 0.5	Increased size <50% between 2 consecutive exams
	2	Present		Add 1	Increased size ≥50% between 2 consecutive exams
Calcification	0	Absent	Calcification size	0	Stable
	1	Doubtful		Add 0.5	Increased volume ≤ 50% between 2 consecutive exams
	2	Present		Add 1	Increased volume > 50% between 2 consecutive exams

The angle of dorsolumbar kyphosis was defined for each mouse at M3, M6, M9, and M12. Using sagittal images of mice spines from full body CT scans, endplate orientations of thoracic and lumbar vertebrae were marked using ImageJ (Rasband, W.S., ImageJ, U.S. National Institutes of Health, Bethesda, MD, United States^1^, 1997–2016). The apical thoracic vertebrae of the rachis were identified and the kyphosis angle defined by the means of (1) the tangent of the cranial endplate of the 4th thoracic vertebra and (2) the tangent to the caudal endplate of the 4th lumbar vertebra, from the apical thoracic vertebra. A MATLAB script (MATLAB R2012b, The MathWorks Inc., Natick, MA, 2000) was used to measure these angles in all mice at each age.

### Murine Tissue Preparation

Bones were fixed overnight at 4°C in 70% ethanol solution and dehydrated in a graded ethanol series. Undecalcified samples were embedded in methyl methacrylate (Merck, Rahway, NJ). Serial sections, 4 μm thick, were cut on a microtome (Polycut E microtome, Leica, Wetzlar, Germany). Series of consecutive sections representative of micro-CT images were stained respectively with toluidine blue (pH 3.8), von Kossa reagent (5% silver nitrate solution, Sigma-Aldrich, St Louis, MO), Masson’s trichrome staining (Sigma-Aldrich, St Louis, MO) and safranin O staining (Weigert’s iron hematoxylin, Sigma-Aldrich, St Louis, MO; Fast Green, Prolabo, Paris, France; and Safranin O solution, Sigma-Aldrich, St Louis, MO).

### Immunohistochemistry

Sections embedded in methyl methacrylate were deplasticized in methyl glycol acetate and rehydrated in a graded ethanol series to pure distilled water. Sections were incubated in a moist chamber for 12 h at 4°C with goat anti-human osteopontin (OPN) antibody (R&D Systems, Minneapolis, MN, United States) diluted at 5 μg/mL in 10% goat serum/PBS-T, or goat anti-human sclerostin (SOST) primary antibody (R&D Systems, Minneapolis, MN, United States) diluted at 5 μg/mL in 10% donkey serum with 1% BSA/PBS-T. Sections were then incubated with peroxidase-conjugated anti-IgG diluted at 1/200 and Donkey anti Goat IgG secondary antibody Texas red (Thermo Fisher Scientific) diluted at 1/1000, respectively. Control incubations to assess non-specific staining involved the same procedures except that the primary antibody was replaced by non-immune serum.

### Statistical Analysis

GraphPad Prism (GraphPad Prism version 6.0 for Mac) was used for statistical analysis using 2-way ANOVA followed by multiple comparison tests. Differences were considered significant at *p* < 0.05. Data are expressed as mean ± *SD*.

## Results

### Enthesopathies and Joint Structural Alterations Increased With Aging in *Hyp* Mice

**FIGURE 2:**
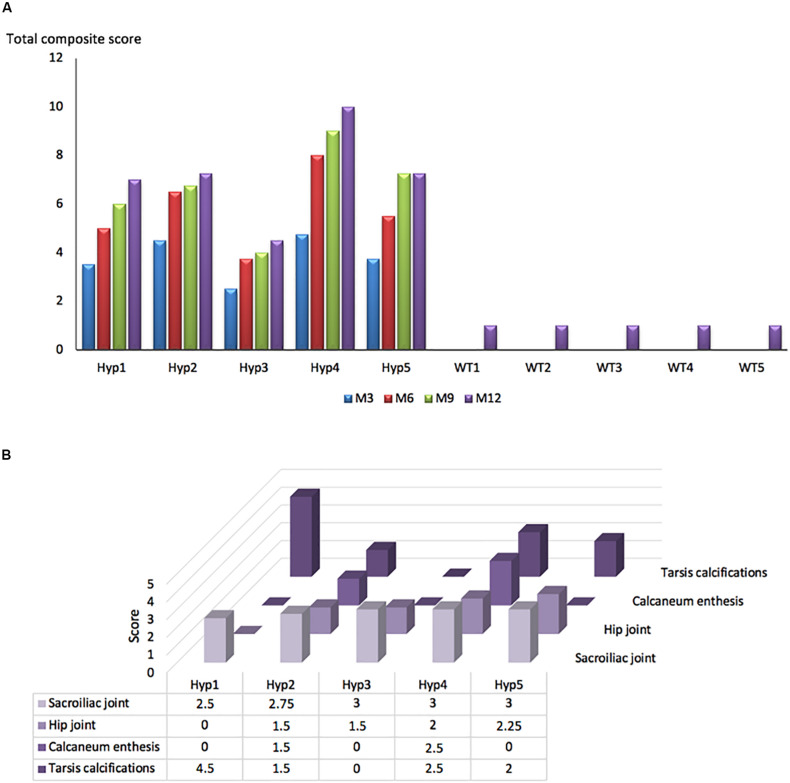
Longitudinal changes in the total composite score in *Hyp* and WT mice. **(A)** A total composite score was established following the scoring grid (Table 1) for each mouse at each time point (M3, 6, 9, 12). Enthesophytes, erosions and ossifications were observed in each *Hyp* mouse and no abnormalities were observed in controls. These lesions were already present at M3, the composite score increasing continuously over time. **(B)** Scores of enthesopathies, erosions, ossifications, and narrowing of joint established following the scoring grid (Table 1) for each *Hyp* mouse at M12. No spinal enthesophytes were observed. The pattern of lesions differed between *Hyp* mice.

A longitudinal follow-up was performed by micro-CT of the skeleton of *Hyp* and control mice. Hip joint alterations, enthesopathies on spine or hind paw, erosion of the sacroiliac joint and periarticular calcification were assessed following the semi-quantitative score detailed in the reading grid in Table 1. Enthesopathies, erosions and osteophytes were detected in all *Hyp* mice from M3 whereas no abnormalities were observed in control mice. The total composite score indicates, overall, a clear increase in structural severity over time in the *Hyp* mice (Figure 2A). On the other hand, the pattern of lesions differed between these mice (Figure 2B). All of them had a high sacroiliac joint score for bone erosion, i.e., a mean score of 2.5 and 2.85 out of 3 at M3 and M12, respectively. Four mice out of five had hip joint osteoarthritis and/or tarsal calcifications with a mean score of 0 and 1 at M3, and 1.8 and 2.1 out of 3 at M12, respectively. Structural damage of sacroiliac and hip joints was strictly symmetrical.

### *Hyp* Mice Developed Peripheral Enthesophytes and Axial Deformation

#### *Hyp* Calcaneal Enthesophytes

**FIGURE 3:**
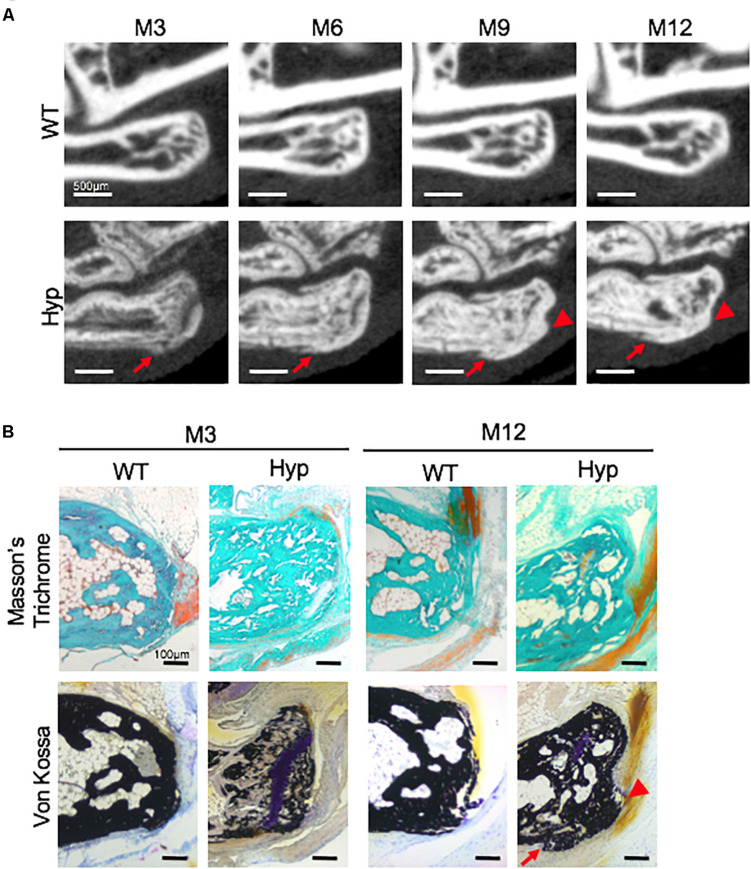
Calcaneal enthesophytes in *Hyp* mice. **(A)** Achilles (red arrowheads) and plantar fascia enthesophytes (red arrows) developed from M3 to M12 in *Hyp* mice (representative micro-CT sections from *Hyp#*4 mouse). In contrast, no enthesis ossification was found in WT littermates (scale bars, 500 μm). **(B)** Masson’s trichrome and Von Kossa staining of undecalcified sections of calcaneal area at M3 and M12 in WT and *Hyp* mice. Von Kossa staining confirmed the cellular expansion of mineralizing fibrochondrocytes into Achilles tendon (red arrowhead) and plantar fascia ligament (red arrow) insertions of the calcaneal tuberosity in *Hyp* mice (scale bars, 100 μm).

Micro-CT follow-up revealed that calcaneal enthesophytes formed early and expanded over time in *Hyp* mice (Figure 3A). Two out of five already had visible calcaneal enthesophytes at M3. Histological sections showed mineralizing fibrochondrocytes expanding into both Achilles tendon and plantar fascia ligament insertions of calcaneal tuberosity in all *Hyp* mice at M12 (Figure 3B).

#### Spine Features

**FIGURE 4:**
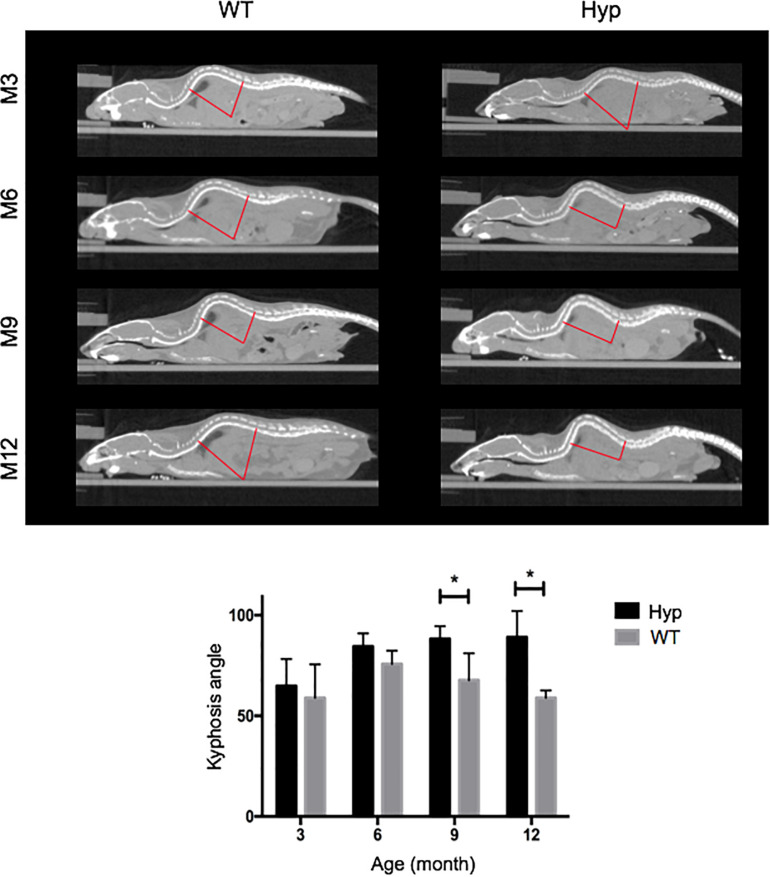
Angle of dorsolumbar kyphosis of mice at M3, 6, 9, and 12. The kyphosis angle was significantly larger from M9 in *Hyp* mice than WT mice (*p < 0.05) (representative micro-CT sections from *Hyp#*2 mouse).

*Hyp* mice developed a significantly larger kyphosis angle from M9 compared to that in WT mice (Figure 4). Although no spine ossifications were observed, we highlighted hypertrophy of the vertebral body on histological staining of *Hyp* mouse vertebrae (Supplementary Figure 1).

#### Hip Joint Features

**FIGURE 5:**
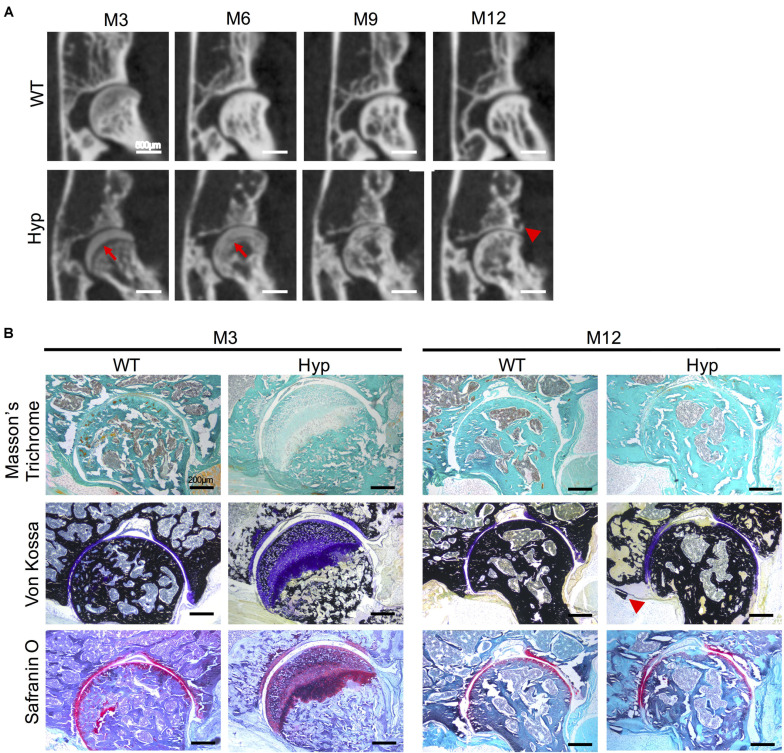
Features of coxofemoral joint of *Hyp* and WT mice. **(A)** Delayed ossification was observed in *Hyp* mice on micro-CT (red arrows). New bone formation was noticeable at M12 in *Hyp* mice (red arrowhead) (representative micro-CT sections from *Hyp#*2 mouse) (scale bars, 500 μm). **(B)** Histological staining confirmed micro-CT observations. High thickness of the non-mineralized cartilage at M3 on Von Kossa staining was evident in Hyp mice which is confirmed by safranin O staining. At M12, the new bone formation was also observable on Von Kossa staining in *Hyp* mice (red arrowhead) (scale bars, 200 μm).

Ossification delay at the femoral head was observed at M3 and M6 (Figure 5A) and confirmed by histological analyses at M3 (Figure 5B). Safranin O staining showed an abnormal thick layer of hypertrophic chondrocytes at M3 in *Hyp* mice, whereas complete ossification of femoral heads was observed in WT mice as confirmed by Von Kossa staining. Micro-CT analyses and Von Kossa staining showed bony outgrowths at the margin of the hip joint in *Hyp* mice at M12 (Figure 5).

### *Hyp* Mice Presented Early Osteoarthritis Features

#### Sacroiliac Joint Features

**FIGURE 6:**
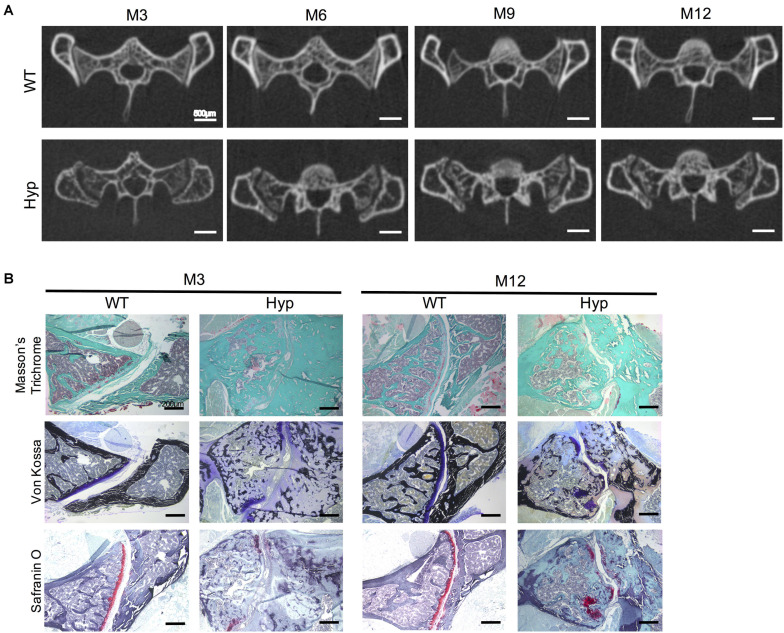
Features of sacroiliac joint of *Hyp* and WT mice. **(A)** Micro-CT analysis showed bone erosion of the sacroiliac joint in *Hyp* mice which appears at early stages and progressed up to M12 (representative micro-CT sections from *Hyp#*2 mouse) (scale bars, 500 μm). **(B)** Masson’s trichrome staining displayed that sacroiliac bone of *Hyp* mice presented a large amount of collagen matrix compared to WT mice. Von Kossa staining showed a weak mineralization (in dark) of the *Hyp* collagen matrix revealing an enlarged osteoid characteristic of features of osteomalacia bone. Safranin O staining revealed high wear of cartilage at M3 in *Hyp* mice (scale bars, 200 μm).

Micro-CT follow-up showed a progressive erosion of the sacroiliac joint with irregular articular surfaces reflecting progressive structural damage in *Hyp* mice compared to controls (Figure 6). These types of damage were present in all the *Hyp* mice as soon as M3 and worsened up to M12. Detailed images obtained from this *in vivo* imaging approach provided a valuable tool to study sacroiliac joint alterations (Figure 6A). Both Masson’s trichrome and Von Kossa staining showed severe osteomalacia at *Hyp* sacroiliac bone and revealed that *Hyp* bone is mainly composed of osteoid matrix at M3 and M12. Safranin O staining showed joint surface damage in *Hyp* mice, characterized by an altered staining of articular cartilage appearing discontinuous along joint surface from M3 and M12 samples (Figure 6B).

#### Periarticular Calcifications

Micro-CT follow up revealed abundant new bone formation on the anterior-inferior side of the tibia in *Hyp* mice, already detectable at M3 and worsening over time up to M12 (Supplementary Figure 2A). This bone formation was confirmed by Masson’s trichrome and Von Kossa staining revealing mineralized collagenous matrix at M3 and M12 (Supplementary Figure 2B).

Periarticular calcifications were also observed in various regions such as the tarsus in the hind paw (Supplementary Figure 3). In contrast, no ectopic bone formation was observed in WT mice even at the end of follow-up.

### Bone Markers Associated With Pathogenic Processes

**FIGURE 7:**
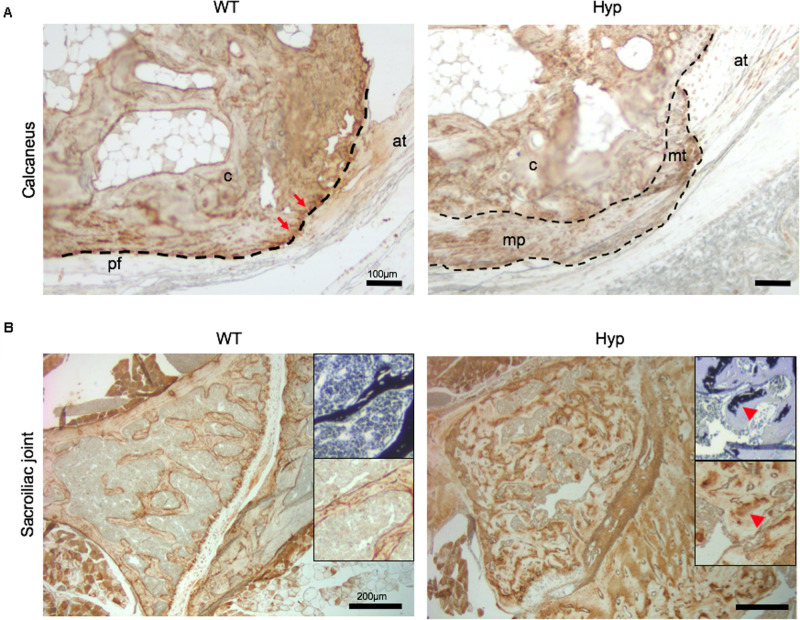
OPN expression in osteoarticular lesions in WT and *Hyp* mice. **(A)** WT calcaneal enthesis showed a strong OPN expression at tidemark (red arrows) between uncalcified and calcified fibrocartilage compared to *Hyp* mice at M12. Cellular expansion of mineralizing fibrochondrocytes of both Achilles tendon and plantar fascia ligament insertions into the calcaneal tuberosity showed faint OPN expression in Hyp mice. pf, plantar fascia ligament; at, Achilles tendon; mt, mineralized fibrochondrocytes of Achilles tendon; mp, mineralized fibrochondrocytes of plantar fascia ligament; c, calcaneal tuberosity; t, tendon (scale bars, 100 μm). **(B)** OPN immunostaining in *Hyp* mice showed a strong expression in sacroiliac joint compared to that in WT mice at M3. The staining was associated with the mineralized matrix rather than the enlarged osteoid matrix (red arrowheads) (scale bars, 200 μm).

**FIGURE 8:**
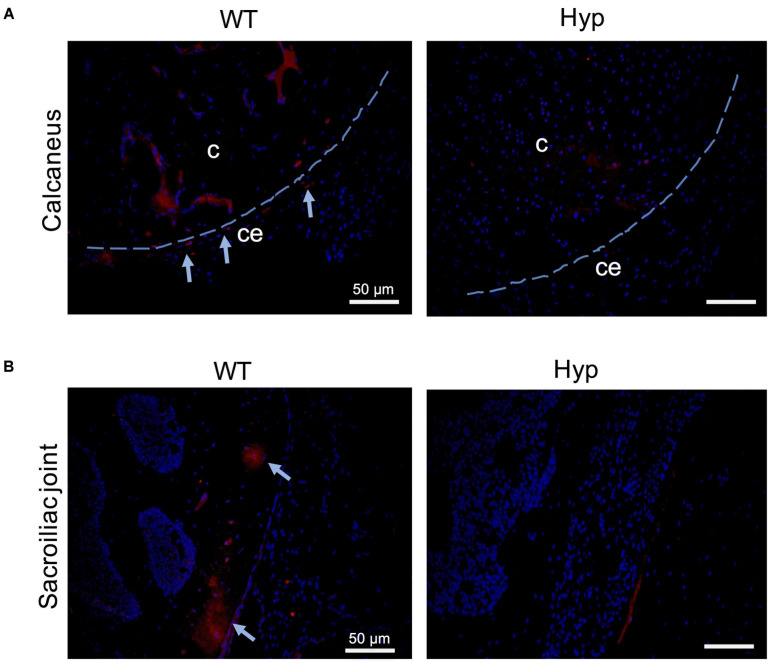
Sclerostin expression in osteoarticular lesions in *Hyp* mice at M3. In WT mice, sclerostin immunolabeled osteocytes and fibrochondrocytes were observed in the calcaneum **(A)** and in the cortical bone of the sacroiliac joint **(B)** (both in the bony part and in the transitional zone between bone and enthesis, indicated with blue arrows). In contrast, very weak expression of sclerostin was seen in these cells in the *Hyp* calcaneal enthesis and sacroiliac joint. c, calcaneus; ce, calcaneal enthesis (scale bars, 50 μm).

In order to investigate the mechanisms underlying abnormal bone formation in XLH, proteins involved in the mineralization process, namely, osteopontin (OPN) and sclerostin, were analyzed in the calcaneus and the sacroiliac joints in control and *Hyp* mice (Moester et al., 2010; Boukpessi et al., 2017). Faint expression of OPN was observed in the *Hyp* calcaneal enthesis, likely associated with the ossification of the enthesis, whereas in WT mice the calcaneum displayed strong OPN labeling (Figure 7A). In the sacroiliac joint, OPN immunostaining appeared stronger than that in WT mice and was associated with the mineralized matrix rather than the enlarged osteoid matrix (Figure 7B). In *Hyp* mice, a very faint expression of sclerostin was observed in osteocytes and fibrochondrocytes in the calcaneum and in osteocytes in the cortical bone of the sacroiliac joint, whereas a strong staining of both cells was seen in WT mice (Figure 8).

## Discussion

In the present study, we report that *Hyp* mice develop enthesopathies, joint ossifications and bone erosions relatively early in life, unlike WT mice. In addition, a progressive worsening of these manifestations was observed over time. These skeletal complications related to early osteoarthritis and enthesopathies phenocopy skeletal features of adult patients with XLH (Figure 1) (Skrinar et al., 2019). Despite their variable severity, these manifestations strongly impair the QoL of adults with XLH similar to the impairment seen in patients with chronic diseases (Che et al., 2016), introducing a novel parameter for the monitoring of therapeutic interventions in XLH. Employing intraindividual follow-up with high-resolution micro-CT and histology on undecalcified bone samples, we documented the natural history of manifestations such as osteoarthritis and enthesopathies in *Hyp* mice. Detailed images obtained by *in vivo* imaging provide a valuable tool for studying structural damage of the sacroiliac joints.

Early features of osteoarthritis are found in up to 85.4% of young adult patients with XLH (Che et al., 2016). In our study, degenerative lesions of the sacroiliac joint were common and developed early in *Hyp* mice. Histological sections revealed that this area was strongly affected in *Hyp* mice, including osteomalacia with accumulation of osteoid matrix and insufficient mineralized tissue. The joints between the sacrum and the ilium are joined by several ligaments and consist of a lower fibrocartilaginous part and an upper ligamentous part. As such, this region is prone to mechanical stress. In the underlying bone under normal conditions, osteocytes are entrapped within the mineralized bone matrix showing a specific morphology and molecular signature. These features allow them to serve as a bone response mechanism for mechanical stress in their microenvironment. Sclerostin, a primarily osteocyte product, is widely considered a key mechanotransduction molecule, whose expression is suppressed by mechanical loading (Moester et al., 2010). Interestingly, we detected a weak expression of sclerostin in *Hyp* mouse sacroiliac joints. This finding could be associated with (1) altered transduction of the mechanical loading due to osteomalacia, or (2) an immature osteocyte phenotype due to the inhibition of the extracellular matrix mineralization. Similar mechanisms have been described when mineralization of osteoid is inhibited by administration of bisphosphonate etidronate (Irie et al., 2008). It is worth noting that a recent study suggests a key role of sclerostin in FGF23 regulation, phosphate metabolism and XLH pathobiology (Carpenter and Ross, 2020).

OPN is an acidic, phosphorylated, calcium-binding, extracellular matrix protein from the small integrin-binding ligand, N-linked glycoprotein (SIBLING) family (Fisher and Fedarko, 2003; Qin et al., 2004), and has been proposed as a key player in XLH pathobiology. OPN mineralization-inhibiting functions are regulated through its degradation by the endopeptidase PHEX for which it is a substrate (Addison et al., 2010; Barros et al., 2013). Indeed, OPN and its mineralization-inhibiting peptides have been shown to accumulate in XLH/Hyp bone matrix, including in periosteocytic lesions (Barros et al., 2013; Boukpessi et al., 2016), and in interglobular dentin (Boukpessi et al., 2010; Salmon et al., 2014), contributing locally to the matrix mineralization defect independent of systemic Pi−regulating factors (Hoac et al., 2020). In the present study, the investigation of the sacroiliac joint showed that, in addition to this abnormal accumulation of OPN in hypomineralized bone regions, this protein was also strongly expressed within the mineralized matrix (Salmon et al., 2014; Boukpessi et al., 2016). Taken together, this might suggest a differential regulation of OPN depending on the mechanical constraints the tissue studied is under.

Enthesophytes are diagnosed in up to 85% of adults with XLH on X-ray and are usually associated with a poor QoL (Che et al., 2016). Here, we performed for the first time a follow-up of enthesopathies by micro-CT in *Hyp* mice over 12 months. Our study allowed the identification of severe calcaneal enthesopathies in 2/5 mice, consistent with the considerable variability observed in clinical manifestations in humans (Martos Moreno et al., 2016). Histological sections showed mineralizing fibrochondrocytes of the calcaneum in *Hyp* mice (Liang et al., 2009; Liu et al., 2018). Interestingly, sclerostin, which is a potent inhibitor of bone formation down-regulated by mechanical loading, was weakly expressed in *Hyp* calcaneal enthesis. Moreover, OPN localization also appeared to be altered in *Hyp* calcaneal enthesis compared to that in WT mice. Our results support the view that enthesis in XLH patients present an abnormal balance of mineralization factors. Enthesis lesions might develop because of additional stimulation such as biomechanical stress (Jacques et al., 2014).

In contrast, we did not observe axial enthesophytes at the spine in *Hyp* mice, although there was spinal curvature deformation. In humans, however, spinal enthesophytes are a major concern in relation to adult manifestations and QoL, being present in ∼64% of adults with XLH (Che et al., 2016). This specific feature of the *Hyp* spine might be explained by the quadruped locomotion, which strongly modifies spinal constraints compared to those in humans, suggesting that axial enthesophyte formation is probably associated with impaired mechanical loading in humans.

Our study widens the prospects of XLH treatments. Specifically, the conventional medical treatment (phosphorus supplementation and active vitamin D analogs) is commonly prescribed from early childhood to the end of growth and seeks to counteract consequences of FGF23 excess (Linglart et al., 2014). Although biological tests to monitor XLH are performed to avoid complications such as hypercalciuria or hyperparathyroidism, to date, there is no consensus on the indications and duration of treatment in adults (Carpenter et al., 2011). This murine model of XLH appears to be a suitable preclinical model for assessing the long-term effect of therapies on adult skeletal manifestations and developing effective preventive strategies.

A recent therapeutic strategy was developed to neutralize FGF23 action using monoclonal anti-FGF23 antibodies in children and adults with XLH (Carpenter et al., 2014; Imel et al., 2019). In *Hyp* mice, repeated s.c. administration of FGF23 antibody rescues the bone and mineral phenotype, including circulating phosphate levels. The effect of anti-FGF23 antibodies on severe XLH manifestations that strongly impact patient QoL remains to be explored. In particular, the *Hyp* mouse model will make it possible to study the local effect of the new drugs on extracellular matrix mineralization defects due to PHEX deficiency (Coyac et al., 2018). In this context, the study of FGF23 involvement in mineralization defect at the tissue level through the Klotho/FGF-R pathway will be all the more important.

In conclusion, we report early and progressive axial deformation and peripheral enthesophytes in *Hyp* mice. This appears to be a relevant model (i) to investigate pathobiological mechanisms underlying development and severity of enthesopathies and joint structural damage, and (ii) for preclinical studies seeking to test the impact of conventional and new therapies on the development of such manifestations. Further, these findings suggest a new paradigm in which mechanical constraints contribute to the development of enthesophytes and bone structural alterations in XLH. This opens avenues for a new field of mechanotransduction-associated pathways in XLH and improves knowledge of the natural history of this disease that ranks alongside causal understanding in importance for prevention and control.

## Data Availability Statement

The raw data supporting the conclusions of this article will be made available by the authors, without undue reservation, to any qualified researcher.

## Ethics Statement

The animal study was reviewed and approved by the Animal Care Committee of French Veterinary Services (DPP Haut de Seine, France: agreement number C-9204901).

## Author Contributions

BS, CM-R, KB, CC, and CB contributed to the design of the experiments. CB, AC, BS, OF, VZ, AB, TS, CR, AL, CM-R, CC, KB, and CB performed and analyzed experiments. KB and CB wrote the manuscript with contributions from all authors. KB and CB supervised the project. All authors reviewed and approved the final version of the manuscript.

## Conflict of Interest

The authors declare that the research was conducted in the absence of any commercial or financial relationships that could be construed as a potential conflict of interest. The reviewer MM declared a past collaboration with several of the authors CC, CB, and AL to the handling Editor.

## Footnotes

1 https://imagej.nih.gov/ij/
