# Supplementary material for: Development of Enthesopathies and Joint Structural Damage in a Murine Model of X-Linked Hypophosphatemia
Source: Front Cell Dev Biol. 2020 Sep 22;8:854. doi: 10.3389/fcell.2020.00854 (PMC7536578; doi:10.3389/fcell.2020.00854)
Supplement: Supplementary file 1 [file Data_Sheet_1.pdf]

Supplementary Material

Supplementary Figures

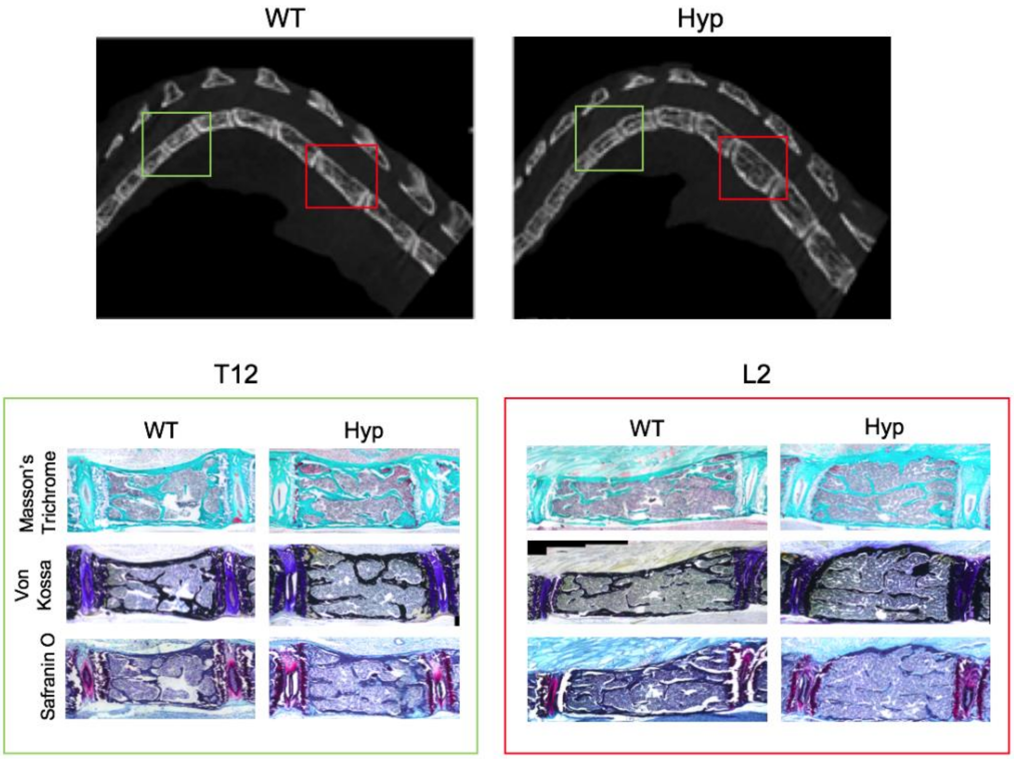

Supplementary Figure 1. Morphological alterations of *Hyp* dorsolumbar vertebrae at M12.
In *Hyp* mice, the lumbar (L) 2 vertebrae located after the apical vertebra was more affected than the thoracic (T) 12 vertebrae, being both shorter and wider compared to those of WT L2 (red square). Masson's trichrome, Von Kossa and safranin O staining confirmed the altered and packed morphology of *Hyp* lumbar vertebrae, with irregular cortical bone and abnormal joints compared to those in WT vertebrae, and showed hypertrophy of vertebral body.

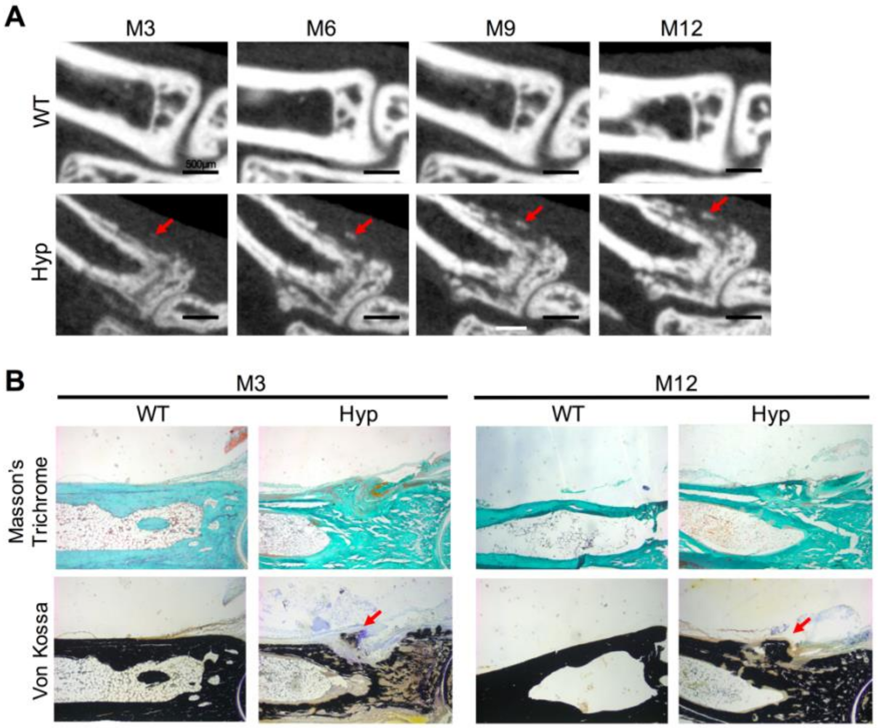

Supplementary Figure 2. New bone formation in *Hyp* mouse tibia.

(A) Micro-CT analysis identified new bone formation in *Hyp* anterior-inferior side of tibia at M3 worsening over time (red arrows) (representative micro-CT sections from *Hyp*#2 mouse). (scale bars, 500 μm).

(B) Masson's trichrome staining revealed an increased collagenous matrix filling the medullary cavity of the tibia in *Hyp* mice compared to WT mice. Von Kossa staining confirmed features of osteomalacia of *Hyp* bone with increased osteoid matrix and new bone formation (red arrows).

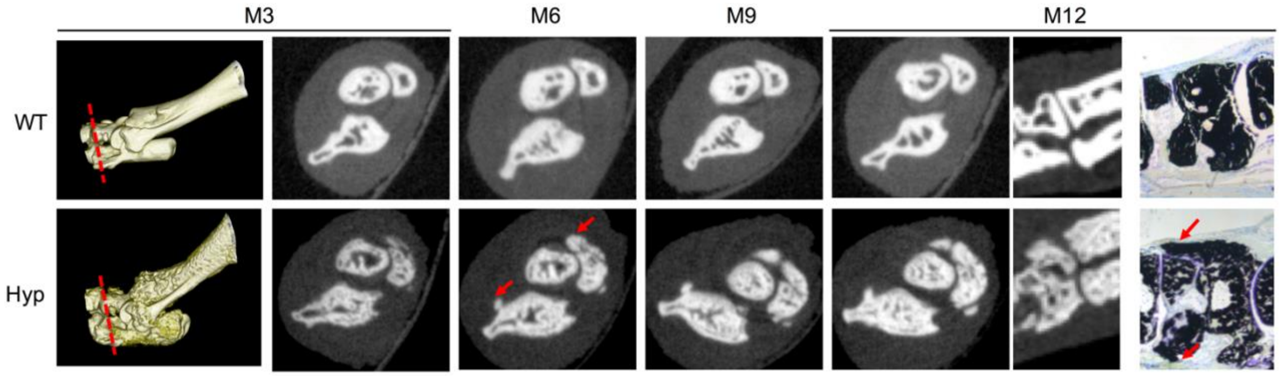

Supplementary Figure 3. New bone formation in *Hyp* mouse hind paws.

Micro-CT analysis showed new bone formation in *Hyp* mouse tarsi in the dorsal and ventral sides of mice paws, from M6 and increasing over time (red arrows) (representative micro-CT sections from *Hyp*#2 mouse). These observations have been confirmed by Von Kossa staining at M12 (red arrows).
